# Sedative defined daily dose: suggestion for a new monitoring tool

**DOI:** 10.62675/2965-2774.20260306

**Published:** 2026-01-28

**Authors:** Danilo Teixeira Noritomi, Walquíria Paula de Melo, Marcos Soares Tavares

**Affiliations:** 1 Ciclo - Eficiência Clínica São Paulo SP Brazil Ciclo - Eficiência Clínica - São Paulo (SP), Brazil.; 2 Diagnósticos da América Barueri SP Brazil Diagnósticos da América - Barueri (SP), Brazil.

Light sedation is considered good practice in the intensive care unit (ICU), as demonstrated across multiple clinical situations.^(1)^ Accordingly, the use of the lowest effective dose of sedatives to maintain patient comfort while ensuring ease of arousal is recommended.^(1,2)^ However, there is no specific recommendation for a single drug or drug class associated with superior clinical outcomes.^(1,3)^ Consequently, comparing sedative consumption among patients across different units or within the same unit over time becomes challenging when preferences for specific sedative agents vary. The defined daily dose (DDD) methodology has appealing characteristics for this task, but it is widely used only in the context of antimicrobial stewardship and rarely for other drugs in the ICU.^(4)^ For intravenous sedatives, only midazolam and dexmedetomidine have World Health Organization (WHO)-assigned DDD values, and these differ substantially from doses commonly used for continuous sedation in the ICU.^(5)^ Accordingly, this potentially useful methodology is not applied as a widespread monitoring tool in sedation practice.

This study aimed to present a novel method to compare the overall use of sedatives across different ICUs, regardless of the individual drug choices.

Between January 1st and December 31, 2022, in a group of 40 ICUs from a private healthcare network comprising ten hospitals, we applied the DDD concept for sedatives in the ICU environment, based on the established definition of DDD and aligned with Society of Critical Care Medicine (SCCM) recommendations. In this way, we created an "adapted DDD" of sedatives in the ICU.

Briefly, the calculation of the number of adapted sedative DDDs is as follows (example in Table 1S - Supplementary Material).

Number of adapted sedative DDDs/1,000 mechanical ventilation (MV)-days = ((daily dose sedative 1/adapted DDD for sedative 1) + (daily dose sedative 2/adapted DDD for sedative 2) + … + (daily dose sedative n/adapted DDD for sedative n)/number of MV-days)) × 1,000.

To calculate the adapted DDD for each drug (Table 1), we used the midpoint of the range, i.e., the arithmetic mean between the lowest and highest values recommended for each drug in the scientific recommendation. We assumed an average weight of the patients based on a hypothetical patient's weight of 80kg.

**Table 1:** Defined daily dose of the main sedatives used

Sedative	Therapeutic range (mg/kg/hour)	Midpoint of the range (mg/kg/hour)	Adapted DDD (mg)
Midazolam	0.02 - 0.1	0.06	0.06 × 80 × 24 = 115mg
Propofol	0.3 - 3.0	1.5	1.5 × 80 × 24 = 2,880mg
Dexmedetomidine	0.2 - 1.2μg/kg/h	0.7μg/kg/h = 0.0007mg/kg/h	0.0007 × 80 × 24 = 1.34mg
Dextroketamine	0.1 - 0.3	0.2	0.2 × 80 × 24 = 384mg

DDD – defined daily dose. Reference doses based on a standardized average weight of 80kg per patient

Throughout the year 2022, using electronic dashboards, we monitored, along with the average Richmond Agitation-Sedation Scale (RASS) score per unit, the value of sedative DDD/1,000 MV-days. We promoted and encouraged best practices for rational sedative use and light sedation, notably: standardized drug dilution, RASS assessment every 4 hours, and discussion of individual sedation goals during multidisciplinary rounds. A target was established for each unit: "< 700 adapted sedative DDD/1,000 MV-days"

A total of 4,791 MV patients were included in the analysis, out of 31,940 ICU admissions. The MV rate was 15%, accounting for 25,041 MV-days. RASS scores and adapted sedative DDD/1,000 MV-days are presented in figure 1.

**Figure 1:**
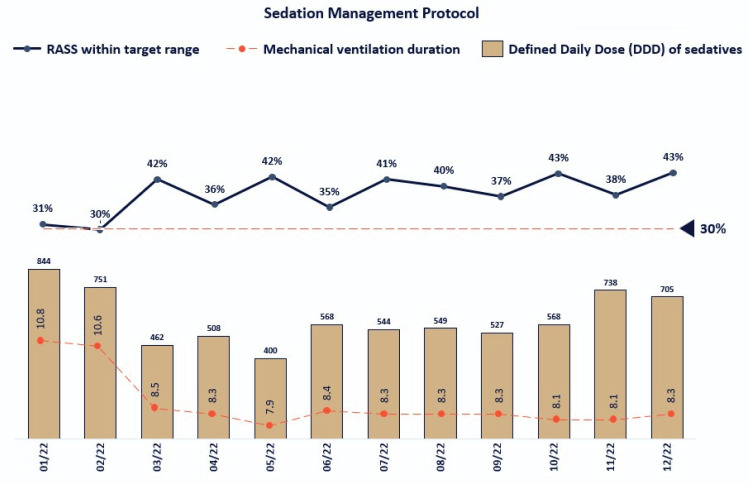
Number of adapted defined daily doses of sedatives per 1,000 mechanical ventilation-days and percentage of Richmond Agitation-Sedation Scale within the target range (Richmond Agitation-Sedation Scale zero to -2) over the 12 months of 2022 in a group of 40 intensive care units.

RASS - Richmond Agitation-Sedation Scale.

The tool has inherent limitations, especially the sensitivity to the chosen reference dose values (DDD). We used the midpoint of the range stated in the most important recommendation of the area, for external validity, but different methodologies could be discussed. Additionally, using a fixed patient weight limits its applicability to ICUs serving populations with markedly different average weights. This limitation can be easily addressed by keeping weight as a modifiable variable. Finally, the accuracy and reliability of this method should be further explored before its widespread adoption.

In conclusion, the adapted sedative DDD tool proved helpful in promoting communication and comparability among ICUs that preferentially use multiple and/or different sedative agents, including different regimens (intermittent or continuous infusion).

## Data Availability

Data is available on demand from referees.
