# Supplementary material for: Sedative defined daily dose: suggestion for a new monitoring tool
Source: Crit Care Sci. 2026 Jan 28;38:e20260306. doi: 10.62675/2965-2774.20260306 (PMC12977208; doi:10.62675/2965-2774.20260306)
Supplement: Supplementary Material [file 2965-2774-ccsci-38-e20260306-suppl1.pdf]

Critical Care Science
https://doi.org/10.62675/2965-2774.20260306

SUPPLEMENTARY MATERIAL

Sedative defined daily dose: suggestion for a new monitoring tool

Danilo Teixeira Noritomi[1], Walquíria Paula de Melo[1], Marcos Soares Tavares[1]

Table 1S - Example of number of sedatives defined daily dose in an intensive care unit with 3 mechanically ventilated patients using estimated and real average weight

Period	January/25				Drug	Adapted DDD for estimated average weight (80kg)	Adapted DDD for actual average weight (74kg)
Patient	DTN	MT	WM	Total (ICU)	Midazolam	115	106
Estimated weight	80	80	80	80	Propofol	2,880	2,652
Actual weight (kg)	86	80	55	74	Dexmedetomedine	1.34	1.2
Mechanical ventilation (days)	7	5	4	16	Dextroketamine	384	354
Infused drugs in the period (mg)				0			
Midazolam	600	0	300	900			
Number of midazolam DDD for actual weight/1,000 MV (days)	NA	NA	NA	530			
Number of midazolam DDD for estimated weight/1,000 MV (days)	NA	NA	NA	489			
Propofol	2,000	1,000	0	3,000			
Number of propofol DDD for actual weight/1,000 MV (days)	NA	NA	NA	71			
Number of propofol DDD for estimated weight/ 1,000 MV (days)	NA	NA	NA	65			
Dexmedetomedine	0		2	2			
Number of dexmedetomidine DDD for actual weight/ 1,000 MV (days)	NA	NA	NA	101			
Number of dexmedetomidine DDD for estimated weight/1,000 MV (days)	NA	NA	NA	93			
Dextroketamine	0	600		600			
Number of dextroketamine DDD for actual weight/1,000 MV (days)	NA	NA	NA	106			
Number of dextroketamine DDD for estimated weight/1,000 MV (days)	NA	NA	NA	98			
Number of DDD sedatives for actual weight/1,000 MV (days)	NA	NA	NA	808			
Number of DDD sedatives for estimated weight/1,000 MV (days)	NA	NA	NA	745			

DDD - defined daily dose; ICU - intensive care unit; MV - mechanical ventilation.

This is an open access article under the CC BY license (https://creativecommons.org/licenses/by/4.0/).
